# Supplementary figures and images for: The relationship between lipoprotein A and other lipids with prostate cancer risk: A multivariable Mendelian randomisation study
Source: PLoS Med. 2022 Jan 27;19(1):e1003859. doi: 10.1371/journal.pmed.1003859 (PMC8794090; doi:10.1371/journal.pmed.1003859)

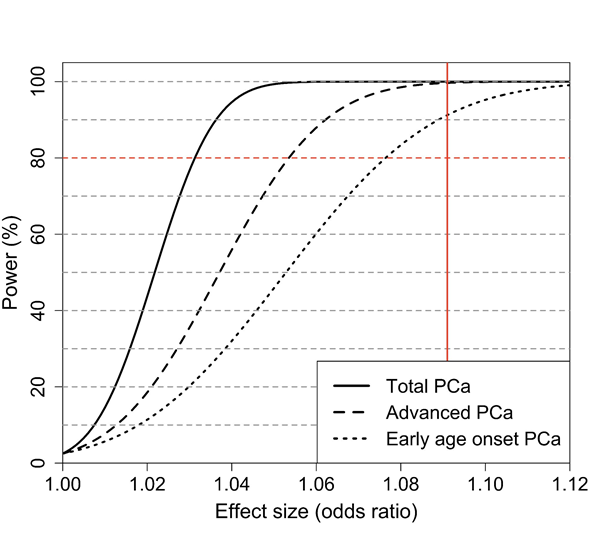

Supplement: S1 Fig — The figure displays the power to detect a significant association on the y-axis against and the true effect size on the x-axis. The different line types indicate the 3 different cases and control numbers for the PCa outcomes. In red, we highlight the observed effect size by the median MR method for total PCa (OR = 1.091; 95% CI = [1.028,1.157]). This power calculation shows that any of the 3 PCa outcomes had a power of 90% or higher to detect an effect of 1.091 or larger. MR, Mendelian randomisation; OR, odds ratio; PCa, prostate cancer. (TIF) [file pmed.1003859.s002.tif]
