# Supplementary material for: The relationship between lipoprotein A and other lipids with prostate cancer risk: A multivariable Mendelian randomisation study
Source: PLoS Med. 2022 Jan 27;19(1):e1003859. doi: 10.1371/journal.pmed.1003859 (PMC8794090; doi:10.1371/journal.pmed.1003859)
Supplement: Attachment — Submitted filename: response_to_reviewers.docx [file pmed.1003859.s026.docx]

**Requests from the editors:**

**Please add this statement to the manuscript's Competing Interests: "VZ is a paid statistical consultant on PLOS Medicine's statistical board."**

**Reply:** We have added this statement accordingly to the Competing Interests.

**In your data availability statement, please provide contact information for data requests (web or email address) from the Practical Consortium. Note that a study author cannot be the contact person for the data.**

**Reply:** We have added the contact information and further details on the PRACTICAL consortium to the data availability statement as follows:

Practical email [PRACTICAL@icr.ac.uk](mailto:PRACTICAL@icr.ac.uk)

**Please remove the ‘Funding’ statement from the title page, in the event of publication this information will be published as metadata.**

**Reply:** We have removed the Funding statement from the title page.

**At this stage, we ask that you include a short, non-technical Author Summary of your research to make findings accessible to a wide audience that includes both scientists and non-scientists. The Author Summary should immediately follow the Abstract in your revised manuscript. This text is subject to editorial change and should be distinct from the scientific abstract. Please see our author guidelines for more information:** [**https://journals.plos.org/plosmedicine/s/revising-your-manuscript#loc-author-summary**](https://journals.plos.org/plosmedicine/s/revising-your-manuscript#loc-author-summary)

**Reply:** We have added the following Author Summary after the Abstract:

# **Author Summary**

## **Why was this study done?**

- Prostate cancer (PCa) is geographically and clinically very heterogeneous and as a result its risk factors may differ according to disease aggressiveness.
- The established PCa risk factors are mainly non-modifiable, which challenge PCa prevention efforts.
- Previous observational research has identified associations between blood lipids and PCa, though results remain inconclusive.
- The aim of this study was to identify evidence for any association between several blood lipids (i.e. LDL, HDL, TG, apoA, apoB and Lp(a)) and total, advanced, as well as early age onset PCa.

## **What did the researchers do and find?**

- The researchers used genetic variants that are known to be associated with each of the blood lipids, to test whether they were associated with any of the three PCa outcomes.
- This Mendelian randomization (MR) analysis can reduce the existence of confounding factors and reverse-causation given that genetic variants are randomly allocated and independently assorted during meiosis. MR provides complementary evidence to observational research.
- This study provided evidence for a positive association between genetically predicted lipoprotein A (Lp(a)) concentrations, but not with other lipids, and risk of total, advanced and early age onset PCa.

## **What do these findings mean?**

- Elevated Lp(a) can play a potentially important role in increasing the risk of PCa.
- It remains however unclear whether Lp(a) is the causal factor given that its pathophysiological mechanisms have not been well-studied.
- These findings provide rationale for further Lp(a) research to understand its functionality and role in PCa, which could lead to repurposing lipid drugs for high-risk individuals that target Lp(a) directly and study their effectiveness against PCa.

**Please open the Discussion with a short, clear summary of the article's findings, before moving on to discussing findings within the context of existing research.**

**Reply:** Thank you for the suggestion. We have added the following paragraph to open the Discussion and summarise our findings:

“Our MR analyses provided evidence that genetically predicted Lp(a) concentration is associated with risk of total, advanced and early age onset PCa. There was little evidence that any of the other lipids (i.e LDL, HDL, TG, apoA and apoB) were associated with PCa outcomes. Specifically, IVs located in the *LPA* gene supported the strongest and most significant Lp(a) associations for total, advanced and early age onset PCa. Given the strong regulation of Lp(a) levels by the *LPA* gene region [28], the latter findings are based on strong instruments with a clear biological function. Adjustment for multiple lipid traits and BMI in the MVMR models, further supported a significant association of genetically predicted Lp(a) on total, advanced and early age onset PCa.”

**Please ensure that the study is reported according to the STROBE guideline, and include the completed STROBE checklist as Supporting Information. Please add the following statement, or similar, to the Methods: "This study is reported as per the Strengthening the Reporting of Observational Studies in Epidemiology (STROBE) guideline (S1 Checklist)."**

**The STROBE guideline can be found here:** [**http://www.equator-network.org/reporting-guidelines/strobe/**](http://www.equator-network.org/reporting-guidelines/strobe/)

**When completing the checklist, please use section and paragraph numbers, rather than page numbers.**

**Did your study have a prospective protocol or analysis plan? Please state this (either way) early in the Methods section.**

**a) If a prospective analysis plan (from your funding proposal, IRB or other ethics committee submission, study protocol, or other planning document written before analyzing the data) was used in designing the study, please include the relevant prospectively written document with your revised manuscript as a Supporting Information file to be published alongside your study, and cite it in the Methods section. A legend for this file should be included at the end of your manuscript.**

**b) If no such document exists, please make sure that the Methods section transparently describes when analyses were planned, and when/why any data-driven changes to analyses took place.**

**c) In either case, changes in the analysis-- including those made in response to peer review comments-- should be identified as such in the Methods section of the paper, with rationale.**

**Reply:** We have submitted the STROBE MR checklist for our manuscript following the guidelines specific for Mendelian randomisation studies (<https://www.strobe-mr.org/>) as supplementary document. The Methods section now transparently describes when analyses were planned, and when/why any data-driven changes to analyses took place including those made in response to peer review comments.

**The paper does not discuss the involvement of PSA levels in relation to the findings. PSA levels of >100ng/mL were used as a criteria for inclusion - if possible, could the authors please comment on how high/low PSA levels affected the analysis or the sensitivity of the screen?**

**Reply:**

Very high PSA levels were only used to define advanced PCa along with information on stage, grade and severity of the disease, as noted in the exact definition in the manuscript. The analysis was based on summary-level data and we did not have access to PSA data at the individual-level. We have added the following clarification to the manuscript how advanced PCa was defined:

“advanced (metastatic or Gleason score (GS) >=8 or PSA>100 ng/mL or PCa death)”

**Comments from the reviewers:**

**Reviewer #1:**

**Ioannidou et al., reported a multivariable Mendelian randomization study on the relationship between blood lipids with prostate cancer risk. They investigated the LDL, HDL, TG, apoA/B, lipoprotein A and GWAS from UK Biobank and PRACTICAL consortium for ~167K individuals. They identified only Lp(a) instead of other lipids was consistently associated with higher total prostate cancer risk in different MR models, the result remained significantly when restricted samples in advanced or early age onset prostate cancer. The paper was well-written, and analysis was carefully performed with extensive sensitivity analysis. See below, some of my questions.**

**Reply:** We are thankful to the reviewer for this pertinent and constructive feedback. Please see our point-by-point reply to your questions below.

**1/ for the study design with case-control setting, it could more informative if a description of control selection can be addressed. How did they match, by age, bmi etc or by propensity score? With current setting of sample size, does it provide enough power to detect significant signals?**

**Reply:** Our Mendelian randomization study is based on a two-sample summary-level data design where genetic associations with the exposure are taken from UK Biobank and genetic associations with prostate cancer and its subtypes are taken from the PRACTICAL consortium. These genetic associations for liability to prostate cancer are based on a large-scale genome-wide association study (<https://www.ncbi.nlm.nih.gov/pmc/articles/PMC6568012/>), which included both cohort and case-control studies adding up in total 79,194 cases and 61,112 controls. We have added a relevant note in the paper that details on the study design can be found in the original GWAS publication. The matching criteria used in the case-control studies shouldn’t affect genetic association estimates. Confidentiality of the data and restricted access to individual-level data prevents us from re-analysing the data for this purpose.

In the following, we provide a post-hoc power calculation for our Mendelian randomization analysis (<https://pubmed.ncbi.nlm.nih.gov/24608958/>), where we set the heritability of the exposures to 24% (as reported for LPa by Sinnott-Armstrong et al. [20]) and the sample sizes of the three different prostate cancer outcomes as follows:

- Total PCa make up to a total of 79,166 cases and 61,106 controls,
- Advanced PCa cases include 15,167 participants and 58,308 controls, and
- Early age onset PCa includes 6,988 cases and 44,256 controls.

The figure below displays power to detect a significant association on the y-axis against the true effect size on the x-axis. The different line types indicate the three different cases and control numbers for the prostate cancer outcomes. In red we highlight the observed effect size by the median Mendelian randomization method for total prostate cancer (OR equal to 1.091 with confidence interval [1.028,1.157]). This power calculation shows that any of the three prostate cancer outcomes had a power of 90% or higher to detect an effect of 1.091 or larger.

We have added this information at the end of Methods and beginning of Results section as follows:

Methods: “Finally, we performed a post-hoc power calculation for our MR analysis [38], where we set the heritability of the exposures to 24% (as reported for Lp(a) by Sinnott-Armstrong et al. [18]).”

Results: “Our power calculation showed that any of the three prostate cancer outcomes had a power of 90% or higher to detect an effect of 1.091 or larger (S1 Fig).”

**2/ with only inclusion of European ancestry in the study, it needs further clarification how to decide whether the participant is EA, what is the cut-off. Should the genetic admixture be included in the MR analysis? Authors mentioned the disparity of the prostate cancer risk with African American has the highest risk. Does the UK biobank have sufficient data from African American to carry the similar MR as the current one?**

**Reply:** We thank the reviewer for this excellent comment. To conduct a two-sample summary-level Mendelian randomization study, we require summary-level data on both the exposure and the outcome from two distinct cohorts. Both UK Biobank (https://pan.ukbb.broadinstitute.org/docs/technical-overview) and the PRACTICAL consortium provide genetic associations for African ancestry, yet at very small sample sizes. UK Biobank includes 6636 individuals with African ancestry (in contrast 420531 individuals are of European ancestry). The PRACTICAL consortium has African ancestry specific summary-level data on genetic association with prostate cancer for 5327 cases and 5136 controls available.

The following power calculation is based on the same parameters as above (heritability of the exposure equal to 24% and a true effect size of 1.091). Here we show power as a function of sample size where the sample size currently available from the PRACTICAL consortium for prostate cancer in African ancestry (5327 cases and 5136 controls = 10463 samples) is highlighted in red. Based on this power calculation we did not perform the Mendelian randomisation analysis now, but we will try to replicate as soon as sample sizes increase to 20000 or larger. This underlines our call for larger transethnic genome-wide association studies, and with initiatives like the RESPOND (https://respondstudy.org/StudyDetails.aspx) we hope that sufficient sample sizes will soon be available.

**3/ IVs selection, it seems there are several criteria of r-square, 0.001 for main analysis, and 0.01 and even 0.4 for sensitivity analysis. Why did authors choose those cut-offs? How much inflations of SNP number was achieve due to such cut-offs? It is necessary to discuss the pros and cons on relaxing the cut-off to 0.4 to include more IVs, as it could increase the power but also with so many features, it could be potentially overfitted the model. Could combination of machine learning with MR better help with IVs selection?**

**Reply:** We thank the reviewer for this excellent comment. We have chosen a cut-off of r-squared < 0.001 for our main analysis which is the strictest cut-off currently used in Mendelian randomization analysis and recommended by the current software packages for clumping (<https://rdrr.io/github/MRCIEU/ieugwasr/man/ld_clump.html>). This threshold included the smallest number of instruments and we have the highest confidence in these results, which we present as the main analysis. Ideally, results from a Mendelian randomization analysis do not depend on the r-squared threshold for clumping. Consequently, we have conducted sensitivity analysis where we set r-squared to 0.01 (Sensitivity analysis 1: including more genetic variants) and to 0.4 (Sensitivity analysis2: according to one previous Mendelian randomization analysis which considered LPa as exposure). The key finding here is that for Total and Early-age onset PCa the results are consistent for main, sensitivity analysis 1 and sensitivity analysis 2, and irrespective of the clumping threshold we detect a significant association of genetically predicted LPa levels with PCa.

The use of machine learning methods for IV selection is a very exciting idea and first approaches (https://doi.org/10.1101/173682, PMID: **34155684,** DOI: [10.1002/sim.9109](https://doi.org/10.1002/sim.9109)) are starting to explore these ideas. Yet, these methods are currently in its infancy and not established for MR analysis or recommended in the MR STROBE guidelines (<https://peerj.com/preprints/27857/>).

**4/ Could authors provide a table of IVs and their statistics to be involved for the main analysis for each lipid, especially Lp(a), do those IVs fall into the LPA gene regions, overlapped with sensitivity analysis 3?**

**Reply:** Summary statistics for all IVs included in our analyses can be found in supplementary tables 10-15. In light of your question, we have added 2 additional tables (S21 & S22 Tables) in the supplement, where we explicitly provide these statistics for the main lp(A) univariable MR, as well as for sensitivity analysis 3. Out of 10 IVs used in the main analysis and 4 IVs used for sensitivity analysis 3, only 1 IV was found to be common (rs4646272).

**5/ for the multivariable MR, can author explore the effect of aspirin use as adjustment to the analysis, as many studies showed aspirin decreases lipids level and advanced prostate cancer risk.**

**Reply:** The reviewer is right that NSAID/aspirin use may confound our results. Unfortunately, we can not act upon this potential limitation by adjusting for aspirin use in the MVMR as we are using only summary-level data and it is not straightforward to define genetic instruments for NSAID/aspirin usage. However, we searched for the 10 IVs used in the main univariable analysis in the Phenoscanner database (P-value threshold=10^-5^) and identified 2 of them being associated with aspirin use. We excluded these 2 SNPs from our IV set and re-ran the univariable analysis on lp(a) and each of the 3 PCa outcomes. This exclusion did not change neither the direction of the estimated lp(a) effect nor did it attenuate the significance of the p-values. We report these findings in S13 Table and have added the following paragraph in the Methods section:

“We reviewed the Phenoscanner database [36,37] (P threshold=10^-5^) for secondary traits associations of the 10 IVs included in the main univariable analyses for Lp(a) and found two that had secondary associations relevant to inflammation and specifically aspirin use. We thereby excluded these 2 SNPs from the main univariable Lp(a) analysis on total, advanced and early age onset PCa.”

**Reviewer #2:**

**The paper uses Mendelian randomization to examine the effects of six blood lipid traits on the risk of prostate cancer. Using data from the UK Biobank and the PRACTICAL consortium, the authors implement both univariate and multivariable MR and identify a link between LP(a) and prostate cancer, but little evidence of a causal effects for the other blood lipid traits.**

**The paper is well written and makes proper use of the relevant statistical methodology. Its conclusions prove to be robust to a number of sensitivity analyses, and the discussion puts the association between LP(a) and prostate cancer into perspective, comparing it with the relevant literature. While reading the manuscript I only had very minor comments, which I will not bother the authors with. Instead, I recommend that the paper is accepted for publication.**

**Reply:** We are very thankful to the reviewer for considering our manuscript and for their extremely positive comments.

**Reviewer #3:**

**This is a well written manuscript focusing on the relationship between blood lipids and prostate cancer risk. Two-sample MR and MVMR analyses illustrated the causality of Lp(a) and increased PCa risk. The authors adopted proper methods and carefully presented their results, and the limitations and advantages were discussed and well addressed.**

**Nevertheless, I have some concerns for authors to respond.**

**Reply:** We are thankful to the reviewer for this pertinent and constructive feedback. Please see our point-by-point reply to your questions below.

**1.	Page 6, first paragraph: The authors run the univariable analysis on all blood lipids for both total and advance Pca, but for early age onset Pca they focused the univariable analysis on Lp(a), why only for Lp(a) and what are the findings for other lipids?**

**Reply:** Thank you for pointing this out. Our analysis began by investigating the role of blood lipids in total PCa. After observing a significant association for lp(a) we decided to see whether this was also the case for advanced PCa, but since we know PCa is clinically heterogeneous we also re-performed the univariable analyses for all lipids. Early age onset PCa was a final investigation after also seeing the lp(a) effect in advanced PCa. As such, we focused this last PCa analysis primarily on lp(a). However, in light of your comment and as we do understand that this may be confusing given our previous analyses on the other PCa outcomes, we also performed the univariable analysis for all lipids and early age onset PCa.

We observed a significant association between LDL and early age onset PCa only through the IVW method (OR = 1.226; 95% CI:1.037-1.451; p = 0.017) and not with the pleiotropy-robust methods, just as we observed for total PCa. However, in the MVMR analysis we performed, LDL was no longer significantly associated with early age onset PCa, thus the association can be interpreted as potential pleiotropy. Similarly, there was an indication for an association between TG and early age onset PCa through the weighted median approach (OR = 1.223; 95% CI:1.041-1.438; p= 0.015), for which the significance attenuated after adjusting for multiple lipid traits in the MVMR.

We have added a table containing the results for all lipids in the Supplement (S7 Table) and we also report any significant findings in the Results section under “Early age onset of PCa”.

**2.	Page 6, last paragraph: Burgess et al identified 43 IVs in their paper [reference 27], but only 28 of them were used in the current manuscript, will bias be induced on the basis of the selective 28 IVs?**

**Reply:** This is a very good point. Let us explain:

Not all 43 IVs were available in both the exposure and outcome datasets (8 missing).

So we continued using the 35 IVs that were available. However, 7 of those failed to reach genome-wide significance (p < 5 × 10−8) and thus we decided to exclude them. As a result, we are basically avoiding weak instruments bias here, by only including genome-wide significant genetic variants. We have added the following clarification to the manuscript:

”Of the 43 IVs used in the paper, 35 IVs were available in both the exposure and outcome dataset. In order to avoid weak instrument bias, we included only 28 genetic variants which were genome-wide significant for Lp(a).”

**3.	Page 7, 3rd paragraph: obesity was used as a confounding factor for lipids and Pca, but lipids including Lp(a) may not be independent of obesity, and this may violate the 2nd assumption of using MR, how do the authors explain this potential bias?**

**Reply:** We thank the reviewer for this excellent point. Obesity may indeed induce genetic confounding and this is why we have performed multivariable MR (MVMR) adjusting for body mass index (BMI). The adjustment for BMI was specifically made in order evaluate assumptions 2 and 3, as MVMR is known to be useful in cases where these assumptions may be violated due to the presence of a known confounding factor (PMID: 30535378, PMCID: [PMC6734942](http://www.ncbi.nlm.nih.gov/pmc/articles/pmc6734942/), DOI: [10.1093/ije/dyy262](https://doi.org/10.1093/ije/dyy262))

Thus, under this scenario, we have adjusted for multiple lipid traits to evaluate assumption 3 and adjust for potential pleiotropy, but we have also adjusted for a possible confounding factor (BMI) in order to estimate the direct effect of lp(a) on PCa which is independent of other lipids and obesity.

**4.	ALT and AST are the established key biomarkers for liver function. Why did the author only select AST in MVMR analysis? What are the impact from ALT? Are there any conflicting findings with ALT? I would suggest the authors to provide relevant results with ALT to enhance their results.**

**Reply:** Thank you for your suggestion. We have additionally performed a MVMR analysis with ALT. Similar to our results from the AST-adjusted model, ALT did not affect the effect size and direction of genetically predicted Lp(a) on PCa, while the significance of the result remained when considering the MR-Egger method. We report the results in the Supplement (Table S12 and have added the following text to the manuscript:

Methods section: “Lp(a) is assembled in the liver [31], whereas liver function/disease has been proposed to influence PCa detection and outcomes [31-32]. Genetic associations for aspartate aminotransferase (AST) and alanine aminotransferase (ALT) were thereby adjusted in a MVMR model including Lp(a) and total PCa. ”

Under Results section: “Results from the additional methods we used, including MR-Egger (S4-S8 Tables), MR-PRESSO and contamination mixture estimates (Table S3), and MVMR analyses adjusting for BMI, AST, ALT and creatinine (S9-12 Tables), can be found in the supplement and were in general in agreement with the main analyses presented in the text below”

Under Results/Multivariable MR section: “The effect size of Lp(a) did not attenuate after adjusting for other genetic confounders we considered (AST, ALT and creatinine) (S10-12 Tables).”

**5. Page 12, last paragraph: I did not find a description for the main findings with regards to Fig 1.**

**Reply:** The last paragraph of the results section refers to Figure 1:

“We compared the multivariable Lp(a) estimates from all the analyses performed on total, advanced and early age onset PCa with the univariable estimates and additional sensitivity analyses through a panel of three distinct forest plots (Fig 1). IVs according to variants in the *LPA* gene (Sensitivity Analysis 3), supported the strongest effect between genetically predicted Lp(a) concentrations and each PCa outcome.”

**6. There are several format errors or typos, such as, capital letters in title, line 232 a redundant "=", etc. Please check the entire manuscript carefully.**

**Reply:** We thank the reviewer for spotting these errors. We have now corrected them in the manuscript.

**7. Please unify the format of p and p-value throughout the manuscript.**

**Reply:** We have unified the format to P.

**Reviewer #4:**

**This study investigates the effects of different lipids on prostate cancer risk using a multivariable Mendelian randomization design. The MR design avoids issues such as unobserved confunding and reverse-causation which are common in observational studies by utilizing predictive genes of different lipids as instrumental variables. The genes are assumed to be randomly allocated to people and thus are independent of both confounders (observed and unobserved) and outcomes (PCa). Therefore, these IVs (i.e. the genes) can help tease out the causal effect of different lipids on PCa.**

**Overall, I think this is a well designed and carefully conducted study. The author carefully cleaned the data and conducted various sensitivity analyses to make sure the results are robust.**

**Reply:** We are thankful to the reviewer for this pertinent and constructive feedback. Please see our point-by-point reply to your questions below.

**Below are specific comments:**

**1.	The authors claim that this study does not require ethics statement. Given the individual-level data used in this study, I am not sure whether that's the case.**

**Reply:** We thank the reviewer for this very important comment. Our study was conducted in a summary-level two-sample Mendelian randomisation design. We take this as an opportunity to clarify this now in the manuscript and we have added in the Abstract, Introduction and Methods that all analysis was conducted on summary-level data.

**2.	The authors mention that there are restrictions of data availability**

**Reply:** As mentioned above, all analysis was conducted on summary-level data. All data from UK Biobank is publicly available from the nealelab (http://www.nealelab.is/uk-biobank). Regarding the outcome data for prostate cancer, while summary-level data on total prostate cancer is in the public domain (http://practical.icr.ac.uk/), data on advanced and early-age onset prostate cancer is restricted access, but available from the PRACTICAL consortium upon application.

We have added the following Data Availability statement to the manuscript:

*“Our study was conducted in a two-sample, summary-level Mendelian randomization design. The exposure data on genetic associations with lipoproteins was provided by the Nealelab (*[*http://www.nealelab.is/uk-biobank*](http://www.nealelab.is/uk-biobank)*). The outcome data on genetic associations with total prostate cancer is available for download from the PRACTICAL consortium (http://practical.icr.ac.uk/), genetic association data on advanced and early-age onset prostate cancer is restricted access, but available from the PRACTICAL consortium upon application (contact: PRACTICAL@icr.ac.uk).”*

**3.	Line 104-105: What is inferred_sex? What is the model and what these covariates are adjusted for? I think you need to describe the GWA data a bit more for reader to understand what these data are and how those data are used in this study.**

**Reply:** We thank the reviewer for asking for further clarification. The GWAS has not been performed by us, but by the nealelab, that has created an extensive resource of GWAS summary-level data on nearly all phenotypes in UK Biobank. We have consequently rephrased this paragraph accordingly to clarify our data sources and specific technical terms, such as inferred_sex, which is sex inferred from the genotype data of an individual:

*“*Genome-wide association (GWA) data for HDL, LDL, TG, Lp(a), apoA and apoB were available from UK Biobank, with information on over 13.7 million single-nucleotide polymorphisms (SNPs) and downloaded from the Neale lab [17]. Model adjustments in this UK Biobank GWAS from the Nealelab included age, age^2, sex (as inferred by genotype), interaction terms for age*sex and for age^2*sex, and the first 20 principal components*..”*

**4.	Line 108: I am not sure what is "heritability estimation from summary statistics". May need a bit explanation for readers who are not familiar with GWA data.**

**Reply:** We thank the reviewer for allowing us to further clarify this part. Heritability estimation from summary statistics (HESS) is an algorithm proposed by Huwenbo Shi (<https://www.sciencedirect.com/science/article/pii/S0002929716301483>) that allows to estimate heritability defined as the proportion of variance explained by the measured genotype. Heritability is an important aspect when performing a Mendelian randomisation analysis, because one of the instrumental variable assumptions requires that the exposure can be predicted by the genetic variants used as instrumental variables. We have rephrased this paragraph accordingly to:

*“*When performing a Mendelian randomisation analysis, it is important that the exposure can be strongly predicted by genetic variants. Heritability estimates for each of the lipid traits were reported by Sinnott-Armstrong et al. [18] based on the HESS algorithm [19] were reported as follows; HDL 36%, LDL 29%, TG 29%, Lp(a) 24%, apoA 31% and apoB 32% indicating strong genetic regulation of all lipid traits considered as exposures a (S1 Table).”

**5.	Line 116: what are these imputation methods? Are you imputing missing values or what?**

**Reply:** Similar as for the exposure data, we also work with existing summary-level data of genetic associations with the outcome, prostate cancer, which was provided by the PRACTICAL consortium. We report in the manuscript important analytical details how the summary-level data that we work with was created, but we agree with the reviewer that these details on the imputation methods used by the PRACTICAL consortium are not necessary and consequently deleted them from the manuscript.

**6.	Line 135: How the p-value of 5*10-8e is determined?**

**Reply:** In genome-wide association studies a *p-*value threshold of 5*10-8e is typically used to define genome-wide significance. Genome-wide significance is based on a Bonferroni correction for 1m independent SNPs (<https://pubmed.ncbi.nlm.nih.gov/18348202/>). This is an established threshold in MR studies which guarantees that genetic variants are strong instrumental variables for the exposure (PMID: **32760811,** PMCID: [PMC7384151](http://www.ncbi.nlm.nih.gov/pmc/articles/pmc7384151/), DOI: [10.12688/wellcomeopenres.15555.2](https://doi.org/10.12688/wellcomeopenres.15555.2)).

**7.	Line 136: What do you mean by removing inconsistencies. What exactly did you do? It could be clearer.**

**Reply:** Harmonization of the data is very important, especially in cases of two-sample MR, as not all studies report the effect and non-effect alleles in the same order. However, in cases of biaellic SNPs, some studies might choose to report the pair of alleles for a SNP on the positive strand, whereas others might report the pair of alleles on the negative strand. So for example, for the same SNP, a pair of A/G alleles can be found on the positive strand and a pair of T/C on the negative strand. Thereby, if for a given SNP one study reported A and G as effect and non-effect alleles, whereas the other study reported T and C as effect and non-effect alleles, we dropped the SNP as no match could be found between the two (PMID: 32760811, PMCID: [PMC7384151](http://www.ncbi.nlm.nih.gov/pmc/articles/pmc7384151/), DOI: [10.12688/wellcomeopenres.15555.2](https://doi.org/10.12688/wellcomeopenres.15555.2).). Upon removing these SNPs, we make sure to have the effect of the SNPs according to the same allele order (harmonization).

We thank the reviewer for allowing us to clarify this in the manuscript and have added more information for the data preparation in the methods section:

“As we combined summary-level data from two sources, we removed inconsistencies in cases where neither the effect nor the non-effect alleles matched for a single SNP between the two datasets. Such cases can occur for a biallelic SNP when one dataset reports the effect of a SNP using a pair of alleles on the positive strand, whereas the other dataset reports the pair for the same SNP on the negative strand [22]. Upon removing these inconsistencies, we harmonised the data so that the exposure and outcome datasets would have the same effect allele. “

**8.	Line 141-142: Why did you focus the UA on Lp(a). Did you pre-plan this or did you peek the data to make such decision?**

**Reply:** Our analysis began by investigating the role of blood lipids in total PCa. After observing a significant association for lp(a) we decided to see whether this was also the case for advanced PCa, but since we know PCa is clinically heterogeneous we also re-performed the univariable analyses for all lipids. Early age onset PCa was a final investigation after also seeing the lp(a) effect in advanced PCa. As such, we focused this last PCa analysis primarily on lp(a). However, in light of your comment and as we do understand that this may be confusing given our previous analyses on the other PCa outcomes, we also performed the univariable analysis for all lipids and early age onset PCa.

We observed a significant association between LDL and early age onset PCa only through the IVW method (OR = 1.226; 95% CI:1.037-1.451; p = 0.017) but not with the pleiotropy-robust methods, in analogy with results for total PCa. However, in the MVMR analysis we performed, LDL was no longer significantly associated with early age onset PCa, thus the association can be interpreted as potential pleiotropy. Similarly, there was an indication for an association between TG and early age onset PCa through the weighted median approach (OR = 1.223; 95% CI:1.041-1.438; p= 0.015), for which the significance attenuated after adjusting for multiple lipid traits in the MVMR.

We have added a table containing the results for all lipids in the Supplement (S7 Table) and we also report any significant findings in the Results section under “Early age onset of PCa”.

**9.	Line 182-184: Bonferroni-correction is known to be very conservative. Did you consider some other corrections which may be more efficient, such as Holm, Benjamini-Hochberg, and Hommel correction, etc.**

**Reply:** We have further included the Holm p-value adjustment in our results and conclusions remain unchanged i.e. in line with the Bonferroni method. We have added the following sentence to the manuscript at the end of the Methods section and included the results in Table 1 :

“We additionally estimated a Bonferroni and a Holm-Bonferroni corrected p-value for the main univariable analyses on total, advanced and early age onset PCa, to adjust for the multiple tests performed on each outcome. The total number of tests is reflected upon the number of different lipids we considered for each PCa outcome”

**10.	Line 267, Table 2: Did you do any correction for the p-values of the MVMR for multiple testing? The p-values are not very small so I am worried that after correcting for multiple testing, the p-values may not be significant anymore.**

**Reply:** We thank the reviewer for this comment. LP(a) has already passed multiple testing correction in the univariable MR analysis we have performed as first step. After this first screen (which adjusted for multiple testing), we focused on LP(a) and move forward with only one hypothesis (LP(a)). Multivariable Mendelian randomisation is known to lack power compared to univariable Mendelian randomisation. We mainly performed multivariable Mendelian randomisation to check the effect strength of LP(a) conditional on the other lipoproteins and if there is any attenuation in effect strength when accounting for other lipids.
